# Cutaneous Manifestations of Systemic Lupus Erythematosus

**DOI:** 10.1155/2012/834291

**Published:** 2012-07-25

**Authors:** Luís Uva, Diana Miguel, Catarina Pinheiro, João Pedro Freitas, Manuel Marques Gomes, Paulo Filipe

**Affiliations:** Faculdade de Medicina de Lisboa, Clinica Universitária de Dermatologia, Avenido Prof. Egas Moniz, 1649-035 Lisboa, Portugal

## Abstract

Systemic lupus erythematosus (SLE) is a multiorgan autoimmune disease of unknown etiology with many clinical manifestations. The skin is one of the target organs most variably affected by the disease. The American College of Rheumatology (ACR) established 11 criteria as a classificatory instrument to operationalise the definition of SLE in clinical trials. They were not intended to be used to diagnose individuals and do not do well in that capacity. Cutaneous lesions account for four of these 11 revised criteria of SLE. Skin lesions in patients with lupus may be specific or nonspecific. This paper covers the SLE-specific cutaneous changes: malar rash, discoid rash, photosensitivity, and oral mucosal lesions as well as SLE nonspecific skin manifestations, their pathophysiology, and management. A deeper thorough understanding of the cutaneous manifestations of SLE is essential for diagnosis, prognosis, and efficient management. Thus, dermatologists should cooperate with other specialties to provide optimal care of SLE patient.

## 1. Introduction

The nosographic concept of lupus erythematosus (LE) includes 3 major subtypes: chronic cutaneous LE, subacute cutaneous LE, and systemic or acute cutaneous LE. Besides these 3 subtypes, other less frequent clinical varieties may occur [1].

Systemic lupus erythematosus (SLE) is a multiorgan autoimmune disease of unknown etiology that can have many clinical manifestations (Table 1). The skin is involved in up to 85% of systemic lupus erythematosus (SLE) cases and may be the only organ involved in cutaneous lupus erythematosus (CLE).

The diagnosis of the cutaneous manifestations of LE is based on clinical, histopathology, and immunohistology of skin lesions. In addition, serum autoantibodies are considered immunologic markers for distinct clinical types of the illness. The Cutaneous Lupus Erythematosus Disease Area and Severity Index (CLASI) is used as a clinical tool that standardizes the way disease activity is described and provides guidelines for identifying a clinical change. This clinical tool quantifies disease activity and damage in cutaneous lupus erythematosus. The activity score is based on the erythema, scale, mucous membrane lesions, and nonscarring alopecia. A recent study gives us a foundation for the practical use of the CLASI in clinical trials as a tool to measure disease severity and responsiveness to therapy [2].

 In 1982, the diagnosis criteria for SLE were published by the American College of Rheumatology (ACR) which were revised in 1997 and are currently used in clinical practice [3]. Undoubtedly useful, mainly for differential diagnosis between systemic LE and other rheumatologic diseases, such criteria are commonly inadequate for some LE subsets. Concerning cutaneous manifestations, the ACR criteria include malar rash, discoid rash, photosensitivity, and oral ulcers. It must be pointed out that the immunologic study does not include the immunohistology of the skin (lupus band test).

## 2. Malar Rash

**Figure 1:**
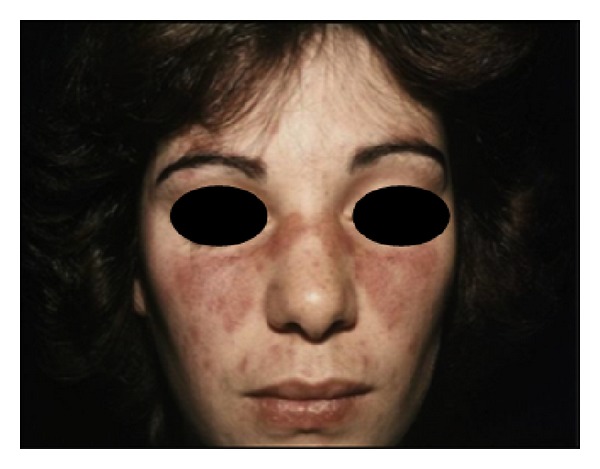
Malar rash.

The first criterion of the ACR is malar rash (sensitivity 57%; specificity 96%), which is characterized by an erythematous rash over the cheeks and nasal bridge (Figure 1). Malar rash is a fixed erythema that typically spares the nasolabial folds. It is a butterfly-shaped or vespertilio rash that can be flat or raised over the cheeks and bridge of the nose. It lasts from days to weeks and is occasionally painful or pruritic.

## 3. Photosensitivity

**Figure 2:**
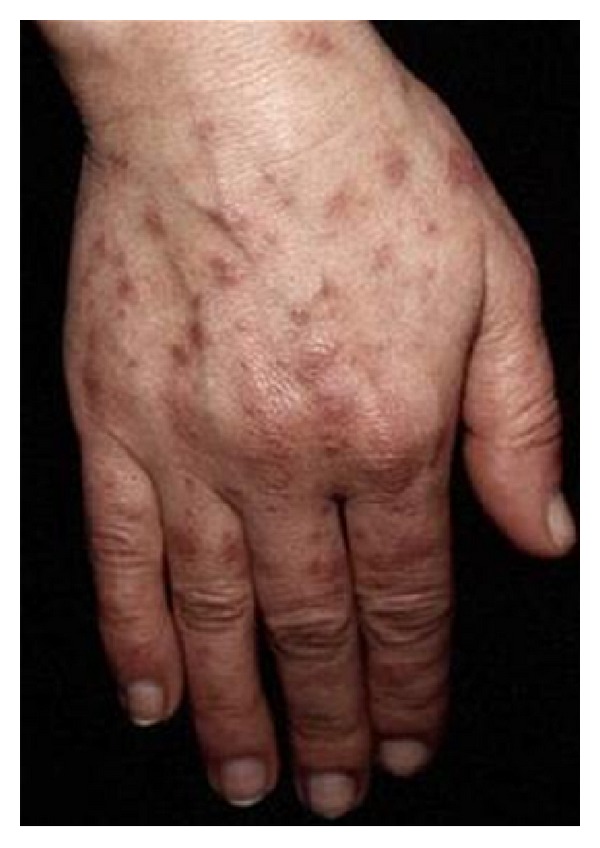
Photosensitive lesions.

The second criterion is photosensitivity (sensitivity 43%; specificity 96%). Exposure to ultraviolet light causes skin rash or other symptoms of SLE flareups. A macular or a diffuse erythematous rash occurs in sun-exposed areas, as the face, arms, or hands and that generally persists for more than 1 day. Sometimes erythematous papules or macules on the dorsal aspects of the hands classically sparing the knuckles are observed (Figure 2).

## 4. Discoid Rash

The third feature may be discoid rash (sensitivity 18%; specificity 59%). Discoid lupus erythematosus (DLE), a chronic dermatological disease, is the most common form of chronic CLE. Lesions may be part of systemic lupus or may represent discoid lupus without organ involvement, which is a separate diagnostic entity.

**Figure 3:**
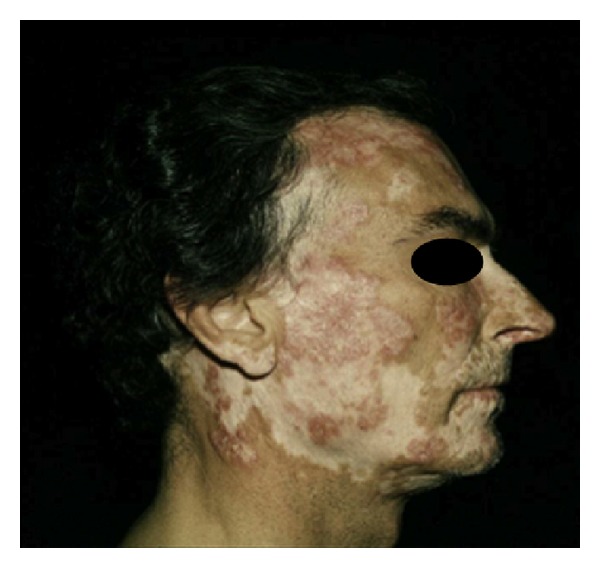
Discoid rash.

Lesions are disc-shaped, erythematous plaques of varying size, and contain areas of follicular hyperkeratoses, which are painful if lifted manually. Disease progression can result in pigmentary changes, permanent, depressed scarring, atrophy, and alopecia (Figure 3). Lesions spread centrifugally and may merge. Although most patients manifest lesional confinement to the head and neck area, a variant termed generalized/disseminated DLE is recognized, for which the minimum criterion is the presence of DLE lesions above and below the neck. Mucosal surfaces may be affected by lesions that appear identical to DLE of the skin or by lesions that may simulate lichen planus. Palms and soles can be also involved, but this occurs in less than 2% of patients [4].

DLE lesions may become hypertrophic or verrucous. This subset is manifested by wart-like lesions, more often on the extensor arms. Hypertrophic lesions of LE must be differentiated from warts, keratoacanthomas, or squamous cell carcinoma. These lesions are more difficult to treat [5]. Lupus panniculitis is a form of chronic CLE that may be accompanied by typical DLE lesions or may occur in patients with SLE [6].

Discoid lupus erythematosus occurs most frequently in women (female/male ratio of 3 : 1) who are 40 to 50 years old [7, 8].

Occurrence of lesions, which may be disfiguring on visible sun-exposed areas, is emotionally devastating and can be added to the psychological burden of the disease. DLE has been reported to have a dramatic negative impact on the patient's quality of life, leading to physical and psychological disability [9, 10].

DLE may occur in patients with SLE, and some patients (<5%) with DLE progress to SLE [11].

Patients with DLE rarely fulfill 4 or more of the criteria used to classify SLE. Serologic abnormalities are uncommon. Serious systemic disease is rare, but when it occurs, patients may develop life-altering sequelae. Malignant degeneration of chronic lesions of lupus erythematosus (LE) is possible, although rare, leading to nonmelanoma skin cancer. Dark-skinned individuals may be more prone to skin cancer because of the lack of pigmentation within the chronic lesion, combined with chronic inflammation and continued sun damage.

Patients may complain of mild pruritus or occasional pain within lesions, but most patients are asymptomatic. Patients with widespread involvement often have hematologic and serologic abnormalities, are more likely to develop SLE, and are more difficult to treat.

## 5. Pathophysiology of Malar Rash, Photosensitivity, and Discoid Rash

The impact of UV irradiation on initial triggering, and on perpetuation of the various cutaneous manifestations of LE, suggests that abnormal photoreactivity is one important factor in LE.

Photosensitivity shows a strong association with the manifestation of all CLE subtypes, and the abnormal reactivity to ultraviolet (UV) light is an important factor in the pathogenesis of both cutaneous and systemic disease. A potentially crucial role in the initiation of the autoimmune reaction cascade has been attributed to UV-induced keratinocyte apoptosis [12]. Interestingly, a significantly higher number of apoptotic nuclei in the epidermis has been described in primary and UV-induced skin lesions of CLE patients compared with normal donors [13]. This is in analogy with the evidence that impair clearance of apoptotic cells may trigger the immune response in patients with autoimmune disorders. Apoptotic cells accumulate in the germinal centers of lymph nodes from patients with SLE, which might be due to impaired phagocytic activity or caused by the absence of tangible body macrophages [14], indicating that apoptotic cells accumulate, and, subsequently, enter late stages of apoptotic cell death including secondary necrosis.

The chromatin nonhistone DNA binding protein high mobility group box one (HMGB1), released during cell activation and death, may also be involved in the inflammatory clearance of apoptotic cells, which justifies the release of HMGB1, detected in the serum of SLE patients as well as an increased expression of HMGB1, was demonstrated in skin lesions of lupus patients. HMGB1 makes easier interaction and uptake (followed by inflammation) by macrophages and dendritic cells through receptor for advanced glycation endproducts and Toll-like receptors 2, 4, and 9 due the connection with nucleosomes and DNA released from apoptotic cells.

Apoptosis or clearance of apoptotic cells has been reported as an important pathophysiological characteristic in autoimmune diseases such as systemic lupus erythematosus, therefore targeting HMGB1 might have an important role on the inflammation control [15].

Nitric oxide (NO), an important regulator of apoptosis, has been implicated in the course of various autoimmune diseases. Interestingly, NO has been shown to protect against UVA-induced apoptosis by increasing Bcl-2 expression and inhibiting UVA-induced upregulation of Bax protein in endothelial cells [16].

In addition, an antiapoptotic role for NO in keratinocytes was suggested after UVB irradiation. Furthermore, UV exposure has also been shown to modulate local production of NO by the constitutively expressed nitric oxide synthase (iNOS). It has also been reported that iNOS is expressed in human skin in the first 2 days after exposure to UVA and UVB [17]. In contrast, in CLE patients, an iNOS-specific signal appeared only 72 h after UV exposure and persisted in the evolving skin lesions up to 1 month, evidencing a delayed and prolonged expression of iNOS in the LE skin. It has further been studied that NO production is increased in patients with SLE, possibly due to the upregulated iNOS expression in activated endothelial cells and keratinocytes [18].

Ultraviolet irradiation leads to release of interleukin-10 (IL-10) by keratinocytes, which may be related with increased autoantibody production and apoptotic damage in skin lesions of LE patients [19]. An interferon-alpha (IFN-*α*) or “type I IFN signature” has been found in patients with SLE. Lesional skin from LE patients has shown a high number of plasmacytoid dendritic cells (pDCs) which are the primary cellular source of IFN-*α*  in LE skin lesions [20]. Interferon-inducible protein-10 (IP-10 or CXCL10), a monokine induced by gamma interferon (MIG or CXCL9) and interferon-a/p-inducible Mx 78 kDa protein (MxA), is downstream surrogate marker for IFN-*α*  expression [21]. IP-10 and MIG recruit CXCR-3 expressing T cells into skin and are abundantly expressed in patients with LE [19].

CJun N-terminal kinase (JNK) is activated by UV radiation [22, 23]. Even low of UVB radiation such as 1 mJ/cm^2^ are capable of inducing JNK activation and the apoptosis of keratinocytes [24]. The expression of iNOS seems also to be dependent upon JNK activity [25].

Naturally occurring CD4^+^CD25^+^ regulatory T cells (T_reg_) have emerged as another important factor in self-tolerance and mechanisms in autoimmune diseases [26]. A decreased number of peripheral T_reg_ were found in SLE patients compared with normal healthy donors and a significant correlation could be detected between the number of CD4^+^CD25^+^ T cells and disease activity [27]. As suggested by Miyara et al., sensitivity of T_reg_ to CD95L-mediated apoptosis could explain the loss of CD4^+^CD25^+^ T cells in patients with active SLE [28].

Recently, a superfamily of small chemotactic proteins has been shown to regulate lymphocyte trafficking of some inflammatory conditions, and it has been demonstrated that UV exposure induces the expression of T cell attracting chemokines [29]. Furthermore, it was shown that CXCR3 ligands, CXCL9, CXCL10, and CXCL11, are overexpressed in patients with CLE. Additionally, it has been reported that the CCR4 ligand TARC/CCL17 is increased in skin and in the serum of patients with CLE [30].

The pathophysiology of cutaneous LE is not clarified, and to find a solution to this problem, appropriate animal models can be helpful to study autoimmune diseases, although no animal model perfectly mimics a human disease.

MRL/lpr mouse is a good model for the spontaneous development of skin lesions similar to those seen in human, but also other models such as transgenic, knockout mice, TCR*α*−/− mice treated with fluorouracil and ultraviolet B light, may contribute to ongoing research, which will shed more light on the pathophysiological relevance of the different cellular and molecular factors *in vivo*, leading to a more complete understanding of the complex events and in SLE [31].

## 6. Treatment of Malar Rash, Photosensitivity, ****and Discoid Rash

Therapy begins with the use of sun-protective measures, including sunscreens, protective clothing, and behavior alteration. Ultraviolet A and B (UVA and UVB) radiations have been implicated in the initiation and exacerbation of skin lesions. As a result, current standard of care includes minimizing sun exposure, and the use of broad spectrum sunscreens. Despite sunscreens are widely used to photoprotect patients with photosensitive lupus erythematosus, standardized controlled studies that can prove their efficacy for this indication have been lacking.

The regular use of sunscreens is beneficial to LE patients because it prevents the UV radiation-induced skin lesions. Effective protection, however, might vary considerably between different sunscreens. A recent study demonstrated that a highly protective sunscreen is able to block the development of UV-induced skin lesions in all patients with the disease. This study confirms that the use of a broad-spectrum (UVB and UVA) sunscreen can effectively protect photosensitive patients with CLE from developing skin lesions.

Topical and intralesional corticosteroids are used for limited disease; however, long-term use may lead to significant side effects, especially on the face. Topical tacrolimus and pimecrolimus have also been shown to be effective on facial lesions in DLE in some patients. In hyperkeratotic lesions, topical retinoids have been reported to be helpful. Recently, topical imiquimod was reported to be effective. When skin lesions are not controlled with topical agents or intralesional corticosteroids, systemic therapy may be indicated.

Antimalarials are the gold standard systemic agents used for DLE. The Food and Drug Administration approved only hydroxychloroquine for the treatment of DLE, but other antimalarials have been used. Most existing regimens have been based on limited clinical experience and empirical data. Recent data says that hydroxychloroquine use is possibly associated with a delay in the development of integument damage and disease activity was associated with a shorter time to integument damage. African Americans have a higher probability of developing integument damage than Caucasians and Hispanics [32].

In 1993, cigarette smoking was suggested to interfere with antimalarial efficacy in treating patients with cutaneous lupus erythematosus (CLE). There are some data which says that cigarette smoking may interfere in a direct manner with the effectiveness of hydroxychloroquine and chloroquine in CLE. It has been hypothesized that the resistance of CLE to antimalarials can be explained by a modification of its metabolism, usually by the induction of cytochrome P450 (in which antimalarial agents are partly metabolized) by the constituents of cigarette smoke. The nonadherence to treatment by smokers could be one of the reasons about how cigarette smoking interferes with CLE treatment. Evidence-based data with long-term followup is required to understand the diminished antimalarial response. Taking into account that smoking negatively affects a number cutaneous conditions, dermatologists are active participants in smoking prevention and cessation [33]. A recent study has shown that there was no significant relationship between cigarette smoking and hydroxychloroquine concentrations, and this is a a strong argument against a direct effect of smoking on hydroxychloroquine metabolism [34].

Up to 30% of DLE subjects are not responsive to the available drugs, and even for those who are responders, long-term use may be precluded by toxicity (e.g., retinal toxicity of antimalarials). As a consequence, no more than 8 months of treatment is recommended. Various studies have shown benefits of thalidomide, with high response rates, even in disease refractory to antimalarials. Adverse events, such as neuropathy, have advised us to not use thalidomide as a first-line agent. Other medications that have been used empirically in subjects that were nonresponders to antimalarial therapy include oral retinoids, clofazimine, dapsone, azathioprine, methotrexate, mycophenolate mofetil, and other cytokine-blocking medications. These medications also have dose-limiting toxicities, including GI side effects, hepatotoxicity, neuropathy, malignancy, and bone marrow suppression [35].

## 7. Oral Ulcers

The fourth criterion of ACR is oral ulcers (including oral or nasopharyngeal ulcers) [36].

**Figure 4:**
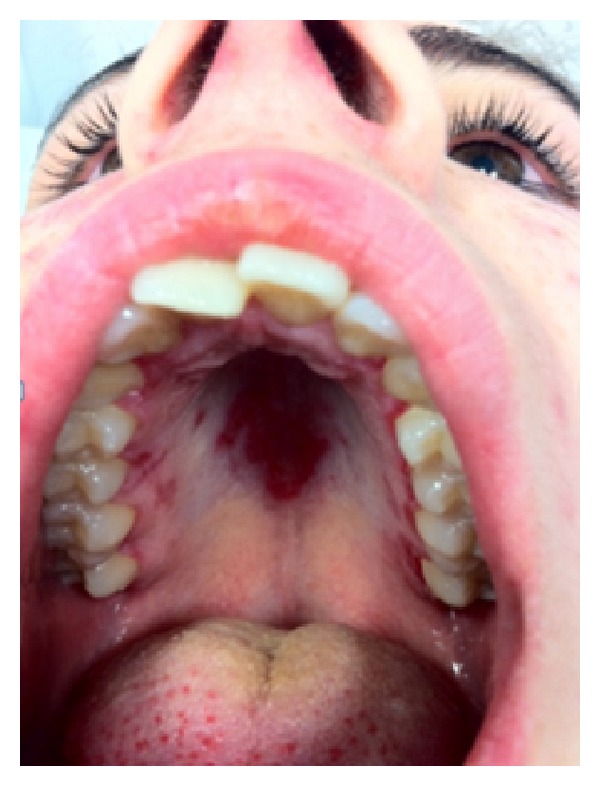
Palatal ulcers.

Lupus should be considered in all patients who experience painless or painful oral (or less frequently nasal or vaginal) ulcers. Palatal ulcers are most specific for SLE (Figure 4).

The prevalence of oral lesions is reported to be 7–52% of patients with SLE [3, 36–48]. Some studies have shown that up to 57% of mucosal lesions were painful whilst other earlier observations stated that up to 82% of oral ulcers observed were painless. This disparity may be due to differences in the type of lesion, whereas erythematous lesions are typically painless, discoid lesions are more often painful. Due to a significant proportion of asymptomatic oral lesions, a careful examination of the oral cavity in all lupus patients must be performed. The relationship between mucosal lesions and systemic disease activity is also nonconsensual. One study specified an association of oral ulceration with clinical systemic activity, although this did not correlate with significant changes in titers of serum complement (C3) or anti-DNA antibodies [38]. It was suggested that patients with oral ulcers have a higher mortality than those without oral ulcers [40], although this has not been confirmed by further studies. It was shown that the overall prevalence of oral lesions was not related to disease activity. However, discoid lesions and ulceration have mainly been seen in patients with active disease.

The buccal mucosa, hard palate, and vermilion border are the locations most frequently involved by lesions [36], which can be three types (discoid lesions, erythematosus lesions, and ulcers) and may coexist [38], leading to oedema and petechiae [36]. Discoid lesions appear as central areas of erythema with white spots surrounded by radiating white striae and telangiectasia at the periphery [38]. Erythematous lesions are often accompanied by oedema and petechial reddening on the hard palate, although they are usually found incidentally as flat macular areas with poorly defined borders [36]. Ulcers tend to occur in crops and are shallow. They are usually 1-2 cm in diameter and in about one-third of patients may extend into the pharynx [38].

No evidence-based recommendations exist for the treatment of oral lupus. A recent large international survey found that mucocutaneous lesions are treated most frequently with antimalarials, steroids and azathioprine are reserved for more severe cases. Thalidomide and cyclosporin are more often used as second-line agents in Europe, whilst North American centers tend to prefer methotrexate [48]. Antileprosy drugs such as dapsone and clofazime have been shown to be beneficial [49, 50].

Preventive dental care is an important issue. Patients have a tendency to consume a diet that promotes dental decay because of impaired taste. The use of chlorhexidine mouthwashes will help to contain periodontal disease and infection. Local treatment of mucous membrane ulcers with hydrogen peroxide gargle, buttermilk gargle, or steroid-impregnated gel may be beneficial. Intralesional injection of corticosteroids may be an option [51]. Suspected infections should be treated with antiviral, antifungal, or antibacterial agents after a swab has been taken for culture and microbial sensitivities.

## 8. Alopecia

Alopecia is an often less specific cutaneous feature of SLE, occurring in about 45 percent of people with lupus at some time during the course of the disease. It often affects the temporal regions or creates a patchy pattern of hair loss. Most frequently, the hair loss occurs at the onset of the illness and may be one of the first symptoms of the disease. When the disease is under control, the hair should grow back. Sometimes there is a rash in the scalp, usually subacute or chronic discoid that interferes with the hair follicle. In this situation, the patient is left with a permanent area of cicatricial alopecia.

The bulge area involvement of the follicles by the inflammation that characterized chronic CLE supports the possibility that damage to the stem cells, which reside on the bulge region, may be one triggering factor to permanent follicles loss. Therefore, the pathogenesis of scarring process in CLE may be explained based on follicular stem cells. Cytokeratin 15 (CK15), a marker of stem cells, has been used to show the bulge region involvement in the scarring process in primary cicatricial alopecia and DLE [52]. Drugs used to treat lupus, such as prednisone and immunosuppressive therapies, also may be responsible of reversible hair loss.

## 9. Subacute Cutaneous Lupus Erythematosus

Subacute cutaneous lupus erythematosus (SCLE) is a photosensitive, nonscarring, nonindurated form of lupus erythematosus. SCLE lesions are related with immunogenetic background that includes the production of Ro/SS-A autoantibodies. Patients who have SCLE skin lesions represent a distinctive subset of LE that has a good prognosis with respect to life-threatening systemic manifestations of LE.

**Figure 5:**
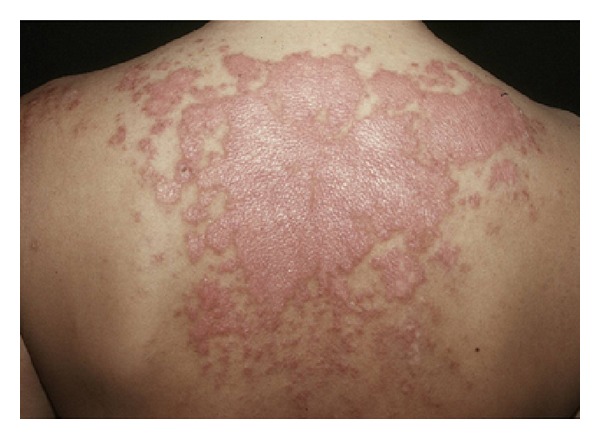
Subacute cutaneous lupus erythematosus.

SCLE skin lesions often initiate as a papular eruption or a small plaque with a slight scaling and may simulate polymorphous light eruption (Figure 5). The enlargement and fusion of these lesions can form either plaques with scaling, in the papulosquamous variant, which may simulate psoriasis or lichen planus, or annular and/or polycyclic lesions, in the annular variant, that may mimic erythema annulare centrifugum.

In addition to papulosquamous variant and annular variant there are unusual variants of SCLE, as tumid lupus erythematosus (TLE) characterized by a cutaneous deeper involvement where little or no scaling is seen. Subacute classification of TLE is controversial, and some authorities have proposed that this variant is better classified as chronic cutaneous lupus erythematosus.

Rowell syndrome, a variant including erythema multiforme-like lesions in association with DLE and chilblains may exist, but it is not sure that this is a distinct entity.

 Sun exposure can induce an exacerbation of the disease, and some patients report worsening each spring and summer. Most patients with SCLE are asymptomatic but mild pruritus could happen in some of those, especially when the lesions occur on the lower extremities.

The etiopathogenesis of SCLE skin lesions is thought to result from different stages such as decline of tolerance/induction of autoimmunity (ultraviolet light, photosensitizing drugs/chemicals, cigarette smoking, infection, psychological stress); susceptibility genetic patrimony (HLA 8.1 ancestral haplotype (C2, C4 deficiency, TNF-*α*-308A polymorphism), C1q deficiency); increasing/maturation of autoimmune responses (high levels of autoantibodies (Ro/SS-A), immune complexes, autoreactive T cells); tissue injury/complaint induction resulting from various autoimmune effector mechanisms (e.g., direct T-cell-mediated cytotoxicity, antibody-dependent cell-mediated cytotoxicity) [53].

Theaim of treatment in subacute cutaneous lupus erythematosus (SCLE) is to improve the patient's appearance and prevent the development of additional lesions. 

Besides sun-protective measures, therapy includes corticosteroids (topical, intralesional) and antimalarials. Treatment with single-agent or combination with aminoquinoline antimalarial will suffice for 75% of SCLE patients. In particular clinical cases, the remaining 25%, have been treated with other pharmacologic forms, as antiinflammatory or systemic immunosuppressive-immunomodulatory therapies, which includes auranofin, dapsone, thalidomide, retinoids, interferon, and immunosuppressive agents [53–56]. 

## 10. Lupus Profundus or Lupus Erythematosus ****Panniculitis

**Figure 6:**
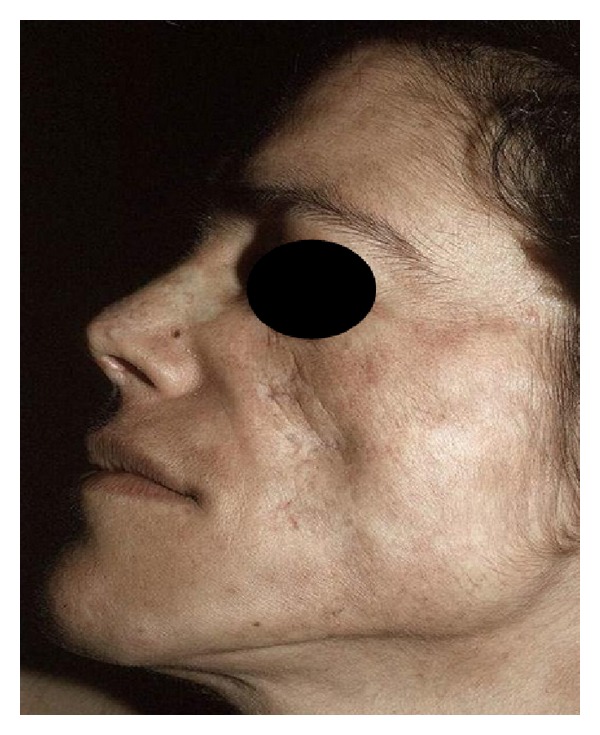
Lupus profundus.

Lupus profundus (LP) isa form of cutaneous lupus erythematosus, which may be the unique manifestation or appear before or after the clinical onset of SLE. Lupus profundus consists of deep brawny indurations or subcutaneous nodules occur under normal or, less often, involved skin; the overlying skin may be erythematous, atrophic, ulcerated, and, on healing, may leave a depressed scar (Figure 6). The most common sites of involvement are proximal extremities, particularly the lateral aspects of the arms and shoulders, thighs, buttocks, trunk, breast, face, and scalp. It can be associated with DLE or SLE. The frequency of occurrence of LP in SLE has been reported to be 2%. The etiology is uncertain. Cytokines and circulating immune complexes may enhance inflammation and hypodermal necrosis observed in LP. Histologically, lymphocytic lobular panniculitis and a characteristic hyaline sclerosis of the adipose tissue are defined.

The most common type of treatment is nonsteroidal anti-inflammatory drugs, or NSAIDS. As an option, antimalarial, drugs, adrenal corticosteroids, and immunosuppressive drugs can be used for treatment as well as chemotherapy drugs for the most severe cases.

## 11. Lichen Planus in LE

LE and lichen planus are usually seen as individual entities. Their overlap comprises patients who have clinical, histological, and/or immunopathological characteristics of both diseases simultaneously. The clinical presentation is a pruritic papular eruption characterized by its violaceous color polygonal shape and, sometimes, fine scale. It is most commonly found on the flexor surfaces of the upper extremities, on the genitalia, and on the mucous membranes. Pruritus is common in lichen planus but varies in severity depending on the type of lesion and the extent of involvement. Hypertrophic lesions are extremely pruritic while oral lesions may be asymptomatic or have a burning sensation, or they may even be painful if erosions are present. Large, annular, hypertrophic lesions and mucous membrane involvement are more likely to become chronic.

Pathophysiologically, lichen planus is thought to be an immunologically mediated disorder.

It has been suggested that CD8+ cytotoxic T cells recognize an unknown antigen associated with the major histocompatibility complex (MHC) class I on lesional keratinocytes and lyse them [57]. T cells and keratinocytes express interferon-*γ*  (IFN-*γ*) and interleukin-6 (IL-6) [58], and T cells also express lymphocyte function-associated antigen-1 (LFA-1).

Mononuclear cells infiltrating the skin, the majority of which are CD8+, as well as basal keratinocytes, express tumour necrosis factor-*α*  (TNF-*α*) and TNF-R1 [59]. Activated T cells secreting IFN-*γ*  induce keratinocyte expression of human leukocyte antigen (HLA)-DR [60], and the presence of epidermotropic T cells correlates with that of HLA-DR-expressing keratinocytes and Langerhans' cells.

The role of chemokines in the pathophysiology of lichenoid tissue reactions regards of recruitment and local activation of cytotoxic Th1 cells and plasmacytoid dendritic cells. Infiltrating CD8+ T cells, as well as keratinocytes, express a variety of different chemokines [61–63]. RANTES (regulated upon activation, normal T cell expressed and secreted) secreted by T cells may trigger mast cell degranulation with consequent release of TNF-*α*, which in turn up-regulates lesional T cell RANTES secretion; such mechanisms may contribute to chronicity of T-cell infiltration and clinical disease [57].

The first-line treatments of cutaneous lichen planus are topical steroids and a second choice would be systemic steroids for symptom control, which leads to a faster resolution. Oral acitretin has been shown to be effective [64]. Many other treatments are used, including mycophenolate mofetil, which efficacy is uncertain, and sulfasalazine in patients with generalized lichen planus [65].

Other cutaneous manifestations related, but not specific, to SLE, include the following:

- Raynaud's phenomenon;
- cutaneous vasculitis;
- periungual telangiectasias;
- urticarial vasculitis;
- livedo reticularis;
- atrophie blanche;
- bullous lesions.

The nonspecific skin lesions, mainly found in active phase of SLE, are characteristic of cutaneous lupus but can also be included on the clinical picture of another disease and it is not possible to establish a histopathological distinction between them [66].

## 12. Raynaud's Syndrome

**Figure 7:**
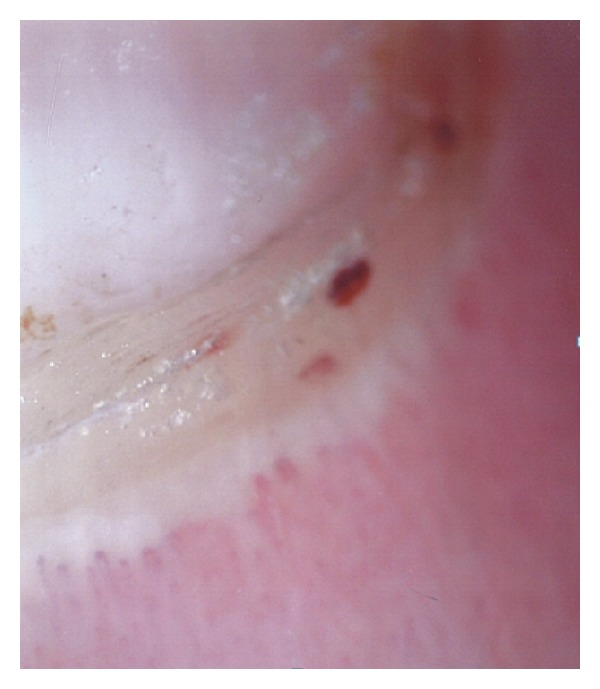
Capillaroscopy.

The Raynaud's syndrome (RS) is an exaggerated vascular response to cold temperature or emotional stress, secondary to identified diseases. This vascular lability is manifested clinically by sharply demarcated color changes in the skin of the digits. Abnormal vasoconstriction of digital arteries and cutaneous arterioles, due to local vascular responses, is thought to be the basis of this disorder [67]. These defective events are reversible, contrary to irreversible causes of ischemia such as vasculitis or thrombosis. It most commonly affects the digits of the fingers but may affect the toes, nose, ears, or even the tongue. Raynaud's phenomenon may be observed with blue, white, and red color change at the distal digital tips. Capillaroscopy can be performed with an ophthalmoscope to search for dilated capillary nailfold loops, giant capillaries, and microhemorrhages (Figure 7).

Management of Raynaud's syndrome involves protecting the fingers and the toes from cold, trauma, and infection.

Unfortunately, patients with autoimmune disorders such as SLE and associated Raynaud's phenomenon do not usually respond well to therapy.

Pharmacologic therapy includes calcium channel blockers, prostacyclin analogues, and pentoxifylline. Key areas of ongoing research include a topical nitroglycerin and a Rho-kinase inhibitor (vasodilator) [68].

## 13. Cutaneous Vasculitis

**Figure 8:**
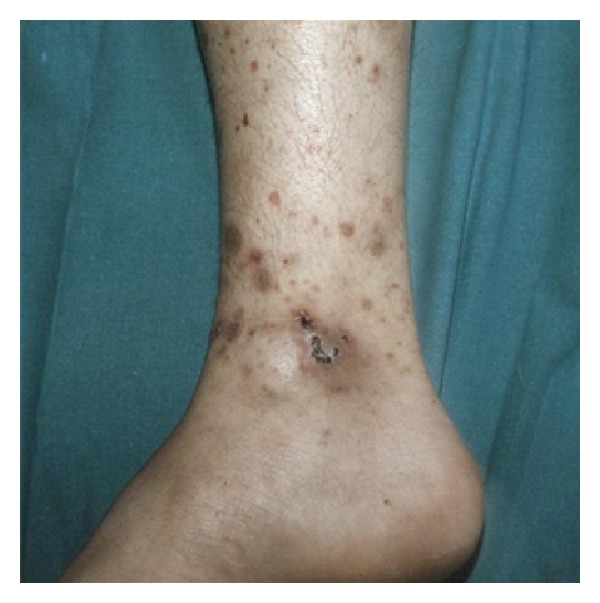
Vasculitis.

Cutaneous vasculitis is presented in a multivariety of morphological lesions such as punctuate lesions, palpable purpura, urticaria, ulcers, papules, erythematosus plaques or macules, and erythema with necrosis that may be self-limiting or relapsing (Figure 8). Cutaneous lesions may be the sole manifestation of the vasculitis or may be part of a systemic involvement.

The most common form of vasculitis seen in LE is a small vessel vasculitis, mediated via circulating immune complexes or by the direct effects of antibodies to cell surface components. Immune complexes are formed in the microvasculature, leading to complement activation and inflammation. Antibody-antigen complexes deposit on the basement membranes of skin. In active SLE, this process has been confirmed by demonstration of complexes of nuclear antigens such as DNA, immunoglobulins, and complement proteins in the skin.

**Figure 9:**
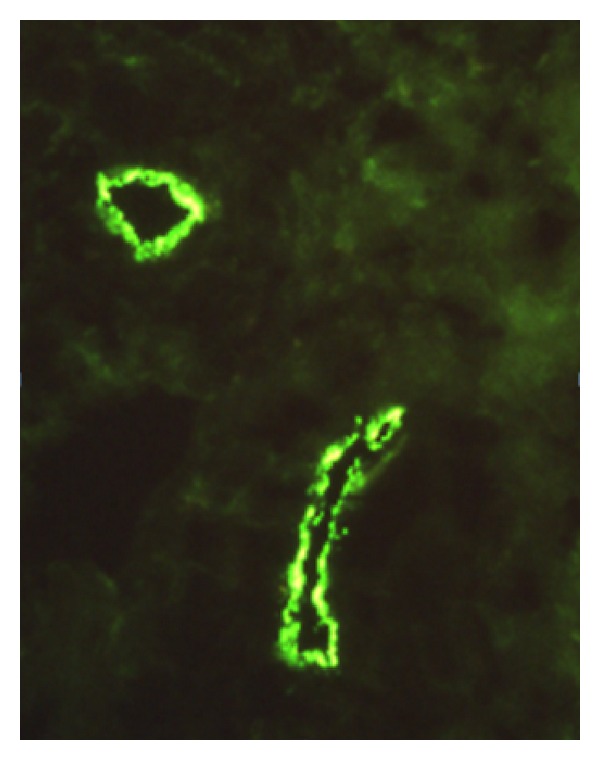
Immune reactant deposits in vessel.

Occasionally, deposition of immune reactants in dermal vessels can be observed, corresponding to vascular involvement (Figure 9). The relative percentages of the different immunoglobulins vary according to authors.

SLE vasculitis is frequently treated with antimalarials, but its discontinuation may result in an SLE flare even in remitted patients. A combination of drugs, plasmapheresis, and intravenous immunoglobulin, along with high-dose steroids and cytotoxicagents, are employed in the treatment of severe SLE vasculitis. Recent data suggests that patients with SLE vasculitis may benefit from a number of autoimmune disease therapies such as switching cytokine responses from Th1 to Th2, and the manipulation of toll-like receptors, chemokines, and FcR receptors. Specific B-cell therapies (e.g., anti-Blys, B-cell depletion) may also emerge as potential treatments for SLE vasculitis.

Responsiveness of cutaneous lupus to rituximab is complex. Discoid lesions do not respond. Acute non-discoid LE and vasculitis in patients with active systemic disease initially improved along with other manifestations. However, some patients switched to a disseminated discoid pattern following B-cell repopulation. This may be explained by the expansion of a T-cell population during B-cell depletion that becomes activated during repopulation. Alternatively transient and incomplete B-cell depletion may alter the pathological B-cell repertoire. The role of B-cells may vary between different patterns of skin disease in SLE and rituximab may not be the most appropriate therapy for all patients. Careful monitoring of the skin is needed when using rituximab in SLE [69].

The last studies about therapy are focused on B-cell targets, T-cell downregulation and costimulatory blockade, cytokine inhibition, and the modulation of complement. Several biological agents have been developed, although with several disappointments in trials [70]. Belimumab has been the only one to be approved in March 2011 by the US Food and Drug Administration (FDA) for the treatment of adult patients with active autoantibody-positive systemic lupus erythematosus who are receiving standard therapy (corticosteroids, antimalarials, immunosuppressives, and NSAIDs) [71]. Other biological therapies proposed for SLE treatment, but not all approved, are, as B-cell targets, Rituximab and Belimumab showing reduction in disease activity; as T-cell targets, Efalizumab (reduces cutaneous SLE manifestations) and Sirolimus (for refractory SLE); as cytokine inhibitors, Infliximab (for lupus nephritis) [70].

## 14. Urticarial Vasculitis

Urticarial vasculitis is an eruption of erythematous wheals that clinically resemble urticarial but histologically shows changes of leukocytoclastic vasculitis. Inversely to urticaria it is usually painful or nonpruritic and typically persists for more than 24 hours. It usually resolves with hyperpigmentation or purpura.

Urticarial vasculitis is a type III hypersensitivity reaction in which antigen-antibody complexes are deposited in the vascular lumina. This reaction activates complement and induces neutrophils chemotaxis. Once activated, neutrophils release proteolytic enzymes, such as collagenase and elastase, damaging the vascular wall. Eosinophils may be involved in the early stages of the vasculitic lesions. Patients with hypocomplementemic urticarial vasculitis are more likely to show autoantibodies to C1q and vascular endothelial cells.

Patients with urticarial vasculitis can be subdivided into two groups, those with normal complement levels and those coursing with hypocomplementemia [72, 73]. The last one is more likely to exhibit systemic manifestations, including constitutional symptoms (fever, malaise, and fatigue), arthralgia, arthritis, serositis, glomerulonephritis, interstitial nephritis, and Raynaud's phenomenon. Angioedema-like lesions are present in 40% of patients, frequently involving the lips, tongue, periorbital tissue and hands [73]. Some patients may present conjunctivitis and episcleritis [74].

Antibodies against C1q are diagnostic markers for hypocomplementary urticarial vasculitis [72–74]. Anti-C1q antibodies were detected in 30% of patients with SLE and 80% of SLE patients with glomerulonephritis [75]. Intravenous methylprednisolone and cyclophosphamide or high-dose oral corticosteroids, colchicine, dapsone, hydroxychloroquine, and low-dose methotrexate have been reported to be effective treatments [76].

## 15. Periungual Telangiectasias

**Figure 10:**
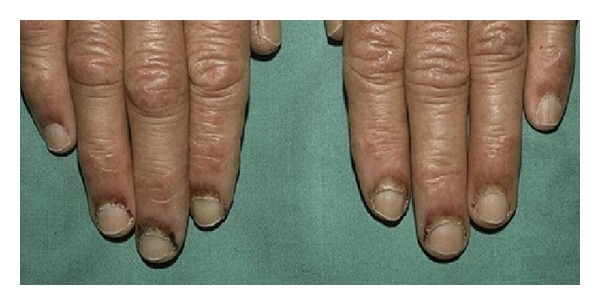
Periungual telangiectasia.

Dilated capillaries of the nailfolds have been found in LE patients (Figure 10). It is better detected by capillaroscopy. Nailfold telangiectasias in SLE patients were associated with anti-U1RNP antibodies [77]. In addition, telangiectasias and erythema of the nailfold were found in 76% of patients who had* *both DLE and SLE, but none in patients with DLE in the absence of SLE, suggesting that this is a rather sensitive indicator for systemic disease activity [78]. Dilated capillary loops dropout are the hallmarks of “scleroderma-pattern” capillaroscopic changes, however, when seen in SLE patients, this pattern of nailfold appears to correlate strongly with Raynaud's phenomenon.

## 16. Livedo Reticularis

**Figure 11:**
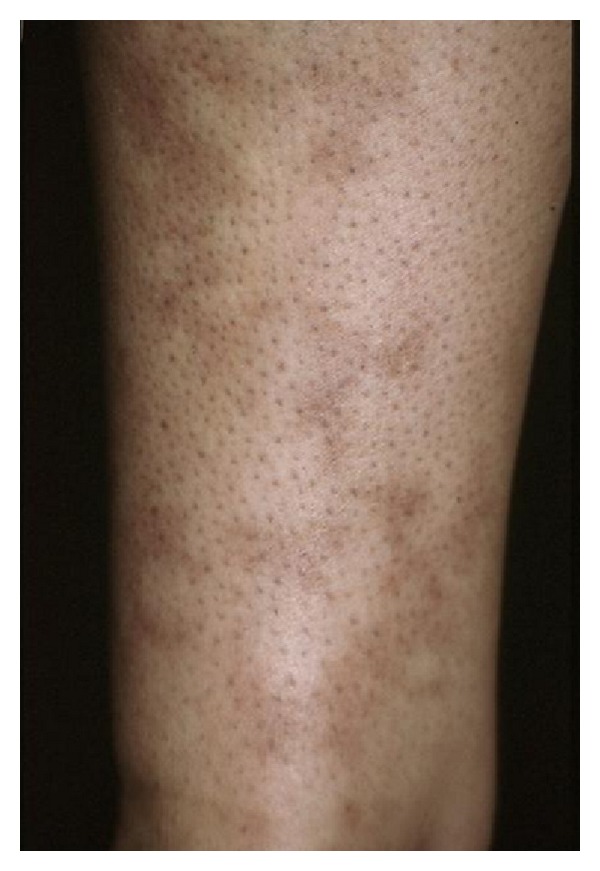
Livedo reticularis.

Livedo reticularis is a common cutaneous reaction consisting of a mottled reticulated vascular pattern that appears like a lace-like purplish violaceous discoloration frequently on the lower extremities (Figure 11) [79]. The discoloration is caused by swelling of the medium veins in skin which makes them more visible. It can be caused by any condition that makes venules swell. The condition may be normal or may be related to severe underlying pathology. It may be aggravated by exposure to cold.

The diagnosis in a patient with livedo reticularis requires a search for associated subcutaneous nodules, retiform purpura, necrosis, and secondary ulceration.

A detailed history can provide valuable information concerning associated diseases such as LES.

Treatment options should be carefully assessed and individualized to each case. Livedo reticularis associated with systemic vasculitis should be treated with corticosteroids and immunosuppressants; doses and combinations will vary according to the clinical condition of the patient and the extent of organ involvement. Serious organ dysfunction requires the use of corticosteroids and cyclophosphamide pulse therapy, and combining low doses of corticosteroids with methotrexate or azathioprine is a good option for maintenance treatment [80].

## 17. Atrophie Blanche

Atrophie blanche is a particular type of scar arising on the lower leg that occurs after a skin injury when the blood supply is poor. One can classify atrophie blanche into primary and secondary types. In the latter such as LES [81].

The clinical presentation is painful petechial, purpuric papules, or hemorrhagic bullae. This last one become, necrotic and forms ulcers, which in turn become atrophic angular scars with hyperpigmentation of the surrounding skin usually on the lower extremities [82, 83].

Atrophie blanche can be the result of circulating immune complexes that are deposited into vessel wall resulting in activation of complement fractions, chemoattraction of neutrophils and fibrin deposition. The release of lysosomal enzymes and reactive oxygen species subsequently lead to secondary vascular damage and inflammatory tissue destruction [84]. Atrophie blanche can also be induced by coagulopathy and this is supported by the fact that fibrins deposit within vessels is the earliest pathogenic change [84, 85].

Systemic corticosteroids, which may be used for recalcitrant cutaneous vasculitis, are ineffective in atrophie blanche. As a matter of fact, prolonged use of corticosteroids in atrophie blanche may result in significant adverse events such as osteoporosis, Cushing's syndrome, hypertension, and glucose intolerance [81]. The most popular regimens include low dose aspirin and dipyridamole which are generally well tolerated and have minimal side-effects [86]. Alternatively pentoxifylline may be used as a rheologic drug consequently improving blood flow [87]. Minidose heparin (SC heparin 5,000 U 12 hourly) has also been reported to be effective in some cases of Atrophie Blanche [88].

## 18. Bullous Systemic Lupus Erythematosus

Bullous systemic lupus erythematosus (BSLE) is an autoantibody-mediated subepidermal blistering disease that occurs in patients with SLE. Blisters and vesicules may arise on erythematous or normal skin and are nonscarring. Lesions occur on sun-exposed or flexural skin. Blistering often parallels flares of SLE involving other organ systems, in particular the kidney. Camisa and Sharma proposed criteria for this distinct subset of vesiculobullous skin lesions occurring in patients with SLE [89]: a diagnosis of SLE based on American College of Rheumatology criteria [90]; vesicles and bullae arising upon but not limited to sun-exposed skin [91]; histopathology compatible with dermatitis herpetiformis [92]; negative indirect immunofluorescence (IDIF) for circulating basement membrane zone antibodies [93]; direct immunofluorescence (DIF) positive for IgG and/or IgM and often IgA at the basement membrane zone. Others have suggested this classification to be revised because of the heterogeneity of clinical and immunohistological presentation [94]. BSLE can be defined as an acquired subepidermal blistering disease in a patient with SLE, in which immune reactants are present at the basement membrane zone on direct, or indirect, immunofluorescence. Direct immunofluorescence microscopy demonstrates immunoglobulin G (with or without immunoglobulin A and immunoglobulin M) deposits at the basement membrane zone (BMZ). Evidence of antibodies to type VII collagen via DIF or IDIF on salt-split skin, immunoblotting, immunoprecipitation, ELISA, or immunoelectron microscopy can be demonstrated.

In patients with BSLE, antibodies directed at the BMZ likely mediate the blistering phenotype by directly interfering with adhesive connections at the dermoepidermal junction and through induction of complement-dependent inflammation that leads to tissue injury and dermoepidermal separation. Proteolytic damage caused by recruited neutrophils contributes to the latter process.

In type 1 BSLE (which accounts for most cases), antibodies against type VII collagen may weaken or block anchoring fibril-mediated connections between the lamina densa of the basement membrane and the papillary dermis. In both EBA and BSLE, antigenic epitopes reside within the NC1 and NC2 domains of type VII collagen, which are localized to the lamina densa and the underlying dermis, respectively. Antibodies recognizing bullous pemphigoid antigen 1, laminin-5, and laminin-6 have also been described in patients with BSLE.

Certain individuals may have a genetic predisposition to develop autoimmunity to BMZ antigens and to SLE. For example, BSLE and SLE are associated with an increased prevalence of the HLA class II DR2 haplotype. The antigen-presenting protein encoded by the DR2-associated DRB1*1501 allele (found in BSLE patients) has been postulated to be involved in presenting type VII collagen epitopes to T lymphocytes.

BSLE occurs in the setting of SLE; thus, ANA test results generally are positive. Anti-dsDNA, anti-Sm, anti-Ro/SS-A, anti-La/SS-B, and anticardiolipin antibodies may also be detected. Other laboratory abnormalities related to SLE can include low levels of complement (i.e., C3, C4, CH50), anemia, leukopenia, thrombocytopenia, proteinuria or cellular casts upon urinalysis, and an elevated erythrocyte sedimentation rate.

All 5 criteria are used to classify type 1 BSLE, whereas only the first 4 criteria are used for type 2 (undetermined location of antigen or dermal antigen other than type VII collagen) and type 3 (epidermal antigen) BSLE [94].

 Dapsone is the initial treatment of choice for BSLE. The response is usually dramatic, with cessation of new blister formation within 1-2 days and rapid healing of existing lesions. Low doses (25–50 mg/day) are often effective, although a higher dosage is sometimes required. Rapid recurrences may occur upon withdrawal of dapsone, with prompt remission after reinstitution of therapy [95]. However, discontinuance of dapsone therapy is usually possible within a year. Prednisone may be effective in patients intolerant to dapsone, have a poor response to dapsone, or require treatment of concurrent systemic manifestations of SLE. Combination therapy with prednisone and dapsone can also be beneficial. Methotrexate, azathioprine, and mycophenolate mofetil represent additional therapeutic options.

Not all blistering eruptions that occur in patients with lupus erythematosus (LE) represent BSLE. Such patients may present with a severe form of acute or subacute cutaneous LE (SCLE) that resembles erythema multiforme (Rowell syndrome) or toxic epidermal necrolysis (TEN).

The eruptions can develop rapidly or evolve over several weeks. In toxic epidermal necrolysis-like acute cutaneous LE, photodistributed diffuse or patchy erythema evolves (usually rapidly) into flaccid bullae (positive Nikolsky sign, unlike BSLE) and widespread sheet-like full-thickness epidermal detachment [94].

The term acute syndrome of apoptotic pan-epidermolysis (ASAP) has been proposed for the TEN-like cutaneous injury pattern that can occur in settings of LE, acute graft versus host disease, pseudoporphyria, and the classic drug-hypersensitivity syndrome. Fas-Fas ligand interactions have been implicated in the massive keratinocyte apoptosis that characterizes ASAP. TEN-like cutaneous LE must be differentiated from drug-induced TEN occurring in a patient with LE. Patients with TEN-like acute cutaneous LE often have significant systemic disease activity (e.g., lupus nephritis, or cerebritis). Extensive eruptions of TEN-like LE require prompt institution of therapy with intravenous immunoglobulin and/or systemic corticosteroids. Less fulminant manifestations of erythema multiforme—like LE can be treated with antimalarials, corticosteroids (topical or systemic) and other agents in the therapeutic armamentarium for LE [96].

## 19. Lupus Band Test

The deposition of immunoglobulin and/or complements at the dermoepidermal junction is a histological feature of LE. Examination of tissue may be done on lesional skin or on nonlesional skin. Nonlesional skin biopsies may be performed on sun-exposed or nonexposed areas. Testing of nonlesional, nonexposed skin is termed the lupus band test.

**Figure 12:**
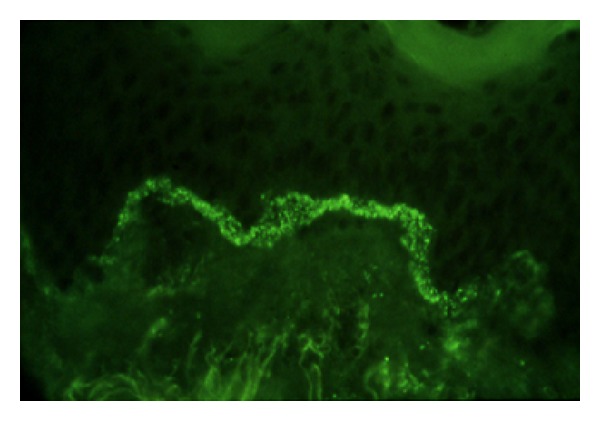
Lupus band test.

By immunohistology, approximately 70% of patients with various subtypes of LE show a positive lupus band test when skin biopsies are performed in normal appearing skin. The normal appearing skin of patients carrying the diagnosis of chronic cutaneous LE, are almost always negative for lupus band test; however, when performed in the cutaneous lesions, lupus band test is positive in about 80% of patients. The fluorescent pattern of dermoepidermal skin deposits of complement or immunoglobulins are either in granules, either in thick band (Figure 12). In some SLE cases, *in situ* ANA deposits were observed. This pattern was first detected by Gilliam in patients with Sharp mixed connective-tissue disease but latter was shown to be not exclusive of the Sharp syndrome, but is also detected in some SLE. It corresponds to an *in situ* epidermis nuclear deposition of the circulating Anti-SSA of those patients.

## 20. Discussion

The American College of Rheumatology (ACR) established 11 criteria in 1982, which were revised in 1997 as a classificatory instrument to operationalise the definition of SLE in clinical trials. They were not intended to be used to diagnose individuals and do not do well in that capacity.

The term “photosensitivity” defined as a rash resulting of an unusual reaction to sunlight by patient history or physician observation is poorly defined, although it is listed as one of the ACR criteria for the classification of SLE. This is an extremely broad definition that can be fulfilled by a variety of other conditions, such as polymorphous light eruption, photoallergic contact dermatitis, and dermatomyositis. In addition, a high disagreement between patient history of photosensitivity and a decreased minimal erythema dose was documented [97]. Concluding that the use of photosensitivity as a classification criterion for SLE remains questionable, at least when it is assessed by patient or physician history according to the ACR criteria. Moreover, the “malar rash,” a further ACR criterion used for the classification of SLE, is often indistinguishable from photosensitivity and, therefore, the two criteria are not completely independent [98].

Up to 73% of patients with systemic LE report photosensitivity, although this correlates poorly with results of phototesting using standardized protocols. Repeated single-patient observations indicate that sunlight may precipitate systemic disease *de novo *or aggravate existing disease. Variation in disease activity related to sun exposure using objective variables has not been shown in large cohort studies; however, two recent studies show that although cutaneous manifestations are more common in the summer months, systemic disease activity is increased in the 3–6 months following maximal potential sun exposure. These observations suggest that summer UV light exposure may lead to flares, after a latency period of several months.

In addition, only 50% of patients with CLE are aware of an adverse effect of sunlight on their disease and, therefore, a negative history of photosensitivity does not necessarily exclude any effect of sun exposure on their disease.

Phototesting with a standardized protocol for UVA and UVB irradiation is an optimal way to evaluate photosensitivity in patients with CLE, confirming that abnormal reactivity to sunlight is an important factor in the pathogenesis of the disease.

Standardized photoprovocation tests with artificial UVA and UVB irradiation are an alternative way to evaluate photosensitivity in patients with CLE demonstrating some differences regarding the various subtypes. However, UV exposure by artificial light sources can trigger systemic organ manifestations [99], therefore, photoprovocation tests should not be performed in all patients with SLE. In the past years, phototesting has been crucial in further characterizing the highly photosensitive subtype intermittent systemic lupus erythematous (ICLE) and has also been shown to be very helpful for the education of patients on photoprotection measures [19]. Therefore, consequent protection against UV light as well as other physical and mechanical injuries are of significant value for the course and prognosis of this disease.

The frequently seen erythematous papules on the dorsal aspects of the hands, typically sparing the knuckles, are completely opposed to the one observed in dermatomyositis.

A revision of ACR criteria is desired, in order to include other dermatologic signs and symptoms, like hand papules sparing the Knuckles. Recently, new classification criteria have been validated. The Systemic Lupus International Collaborating Clinics (SLICC) is an international group, which dedicates their work to the clinical research of SLE. A large set of patient scenarios rated by experts was experimented successfully for the new SLICC classification criteria. They require the presence, at least, of one clinical criterion (nonscarring alopecia is included) and one immunologic criterion for a classification of SLE. Despite that, the diagnostic of nephritis compatible with lupus by biopsy (in the presence of SLE antibodies) is enough for classification. SLICC classification criteria have more sensitivity, but not specificity, than the revised ACR criteria. ACR and SLICC have, statistically, a similar performance. Patients without antibodies or low complement, the hallmark of SLE, cannot be classified as having SLE. The SLICC classification criteria are an alternative for SLE clinical care and research [100].

The lupus band test as an immunologic test for Lupus patients can also be a helpful instrument for differential diagnosis between DLE and SLE.

## Figures and Tables

**Table 1:** Cutaneous manifestations of SLE.

(1) Malar rash	
(2) Discoid LE (DLE)	
(a) Localized DLE	
(b) Generalized DLE	
(3) Photosensitivity	
(4) Mucosal DLE	
(a) Oral DLE	
(b) Conjunctival DLE	
(c) Nasal DLE	
(d) Genital DLE	
(5) Subacute cutaneous lupus erythematosus	
(6) Alopecia	
(7) Lupus panniculitis/lupus profundus	
(8) Lichenoid DLE (LE/lichen planus overlap)	
(9) Small vessel cutaneous leukocytoclastic vasculitis secondary to LE	
(a) Dependent palpable purpura	
(b) Urticarial vasculitis	
(10) Secondary atrophie blanche	
(11) Periungual telangiectasias	
(12) Livedo reticularis	
(13) Raynaud's phenomenon	
(14) Bullous lesions (BSLE)	
